# Consumer Perceptions of Artificial Sweeteners in Food Products, Consumption Frequency, and Body Mass Index: A Multivariate Analysis

**DOI:** 10.3390/nu17050814

**Published:** 2025-02-27

**Authors:** Branka Jurcevic Zidar, Zlatka Knezovic, Ajka Pribisalic, Sanja Luetic, Katarina Jurcic, Nina Knezovic, Davorka Sutlovic

**Affiliations:** 1 Teaching Institute for Public Health, Split-Dalmatia County, 21000 Split, Croatia; branka.jurcevic.zidar@nzjz-split.hr (B.J.Z.); zlatka.knezovic@nzjz-split.hr (Z.K.); sanja.luetic@nzjz-split.hr (S.L.); katarina.jurcic@nzjz-split.hr (K.J.); nina.knezovic@nzjz-split.hr (N.K.); 2 Department of Health Studies, University of Split, 21000 Split, Croatia; dsutlovic@ozs.unist.hr; 3 Department of Public Health, School of Medicine, University of Split, 21000 Split, Croatia; 4 Department of Applied Pharmacy, School of Medicine, University of Split, 21000 Split, Croatia

**Keywords:** artificial sweeteners, foods, consummation, multivariate analyses

## Abstract

Background/Objectives: Artificial sweeteners are commonly used food additives that provide sweetness without calories. Once considered harmless due to their lack of metabolism, recent studies suggest that they may have unintended effects, potentially stimulating appetite and increasing food intake, leading to weight gain. This study aimed to assess consumer perceptions of artificial sweeteners in food, examine consumption frequencies of products containing them, and explore their potential influence on body mass index. Methods: A cross-sectional study was conducted using two voluntary and anonymous surveys administered via Google Forms. Results: The study included 649 participants: 324 parents of preschool and school-aged children and 325 university and secondary school students. A substantial proportion of parents (59.3%) recognized artificial sweeteners as common sugar substitutes in beverages like juices, soft drinks, and protein drinks. Awareness was notably higher among students (88.9%). While most participants held a negative attitude toward artificial sweeteners, their awareness and engagement with food label reading were low. Multivariate linear regression identified significant associations: Male gender (β = 1.17, *p* < 0.001) and older age (β = 0.42, *p* < 0.001) were associated with higher BMI. Additionally, participants who rarely or never consumed carbonated soft drinks had a lower BMI (β = −1.48, *p* = 0.039), while those who occasionally consumed snacks had a higher BMI (β = 0.51, *p* = 0.039). Conclusions: This research underscores the urgent need for public health initiatives addressing misconceptions, raising food label reading practices, while encouraging healthier consumption habits through educational campaigns. Additionally, the study’s insights will help assess the potential cumulative health impacts of artificial sweetener intake.

## 1. Introduction

Food fulfills both a physiological need and a pleasurable function, with taste playing a crucial role in dietary choices. Reducing sugar content often alters the sensory properties of certain foods, leading both consumers and food producers to seek alternatives such as artificial sweeteners. These additives, which vary in chemical composition and sweetness intensity, are widely used to impart sweetness while maintaining low caloric content. Currently, the European Union approves 17 artificial sweeteners and their combinations for use in food products [1]. Their use is regulated under European Parliament and Council Regulation (EC) No 1333/2008 on food additives, which specifies permitted food categories and maximum allowable levels [1].

Although artificial sweeteners were introduced into the food industry in the 1800s, their widespread use surged in the 1990s, coinciding with the rise in obesity and increasing concerns over excessive sugar consumption [2,3]. Initially marketed as a healthier alternative, particularly for individuals with diabetes or those managing their weight, artificial sweeteners gained popularity due to their low-calorie nature. As scientific research increasingly linked high sugar intake to chronic diseases, artificial sweeteners became a common ingredient in various food products. Today, commonly used artificial sweeteners include acesulfame-K, aspartame, cyclamate, saccharin, sucralose, and neotame, often used alone or in combination [3].

Regulatory agencies consider most artificial sweeteners safe with no harmful effects, as they are either not metabolized by the human body or broken down into naturally occurring components [4]. However, emerging evidence suggests potential adverse metabolic effects, including an increased risk of weight gain, insulin resistance, and cardiovascular disease [5,6]. Some studies indicate that artificial sweeteners may trigger cravings for sugar by stimulating appetite due to reduced caloric value, potentially leading to higher overall food consumption, weight gain, and glucose intolerance [7,8]. Furthermore, artificial sweeteners have been associated with an increased risk of type 2 diabetes, premature mortality, and gastrointestinal tract alterations [3,9,10,11], as well as a risk of cardiovascular disease, hypertension, and stroke [12,13]. It is important to note that research in this area is complex, and further investigation is needed to fully understand these associations.

The health effects of artificial sweeteners remain a subject of ongoing debate, with research yielding contradictory findings. Despite efforts to reduce childhood obesity by replacing sugar with artificial sweeteners, the long-term impact of these substitutes on metabolic pathways involved in energy balance and appetite regulation remains uncertain. Recent studies indicate a rising trend in childhood overweight and obesity, both globally and within the Republic of Croatia, particularly in coastal regions where traditional Mediterranean dietary habits are being replaced by processed and sugar-sweetened foods [14]. Increased soft drink consumption has been identified as a major contributor to this shift. Given the limited research on artificial sweeteners in Croatia and the lack of data on their consumption and awareness in specific populations, this study aims to bridge this gap by analyzing the dietary behaviors of families in Split, a major urban center.

Therefore, this study aims to assess the perception and consumption of artificial sweeteners among parents, children, and adults, including university and secondary school students. Given that artificial sweeteners are not only present in beverages but also in “low-fat” and “high-protein” food products—often marketed as healthy choices and popular among younger populations—this study further investigates how these products may influence body mass index (BMI). This study was conducted in the City of Split, Croatia, where childhood obesity trends suggest the need for a better understanding of dietary behaviors and artificial sweetener intake in this population. The results of this research will provide insight into consumption patterns and awareness of artificial sweeteners among parents, children, and adults in Split, Croatia, addressing a gap in region-specific dietary research.

## 2. Materials and Methods

### 2.1. Study Design

This cross-sectional study was conducted in March and April 2024, during which two voluntary and anonymous surveys were administered to participants via Google Forms. The sample size was obtained using online calculators available at http://www.raosoft.com/samplesize.html or https://www.calculator.net/sample-size-calculator.html (accessed on 15 January 2024). Calculations were performed separately for each survey, assuming a 95% confidence level and a 5% margin of error.

The sample calculation for the survey targeting parents of preschool and elementary school-aged children was based on a target population size of 2000 children. This figure was derived from enrollment data, including 1400 children attending two urban kindergartens and 600 children enrolled in an elementary school included in the survey. The kindergartens were selected through discussions with their directors, who expressed willingness to collaborate, while the elementary school was chosen through direct researcher contact and the voluntary participation of teachers and parents who helped distribute information about the survey.

The sample calculation for the survey targeting university and secondary school students had a target population size of 1950 participants, based on the number of students invited to participate. This included 270 medical students (1st, 2nd, and 6th years) and 30 pharmacy students (4th year) from the Faculty of Medicine in Split, 500 students from the University Department of Health Studies (undergraduate and graduate programs), 600 students from other disciplines (natural sciences, technical sciences, and social sciences), 250 senior secondary school students from two grammar schools, and 300 senior students from a vocational and health school.

Therefore, using the online calculators, the required sample sizes were determined to be 323 participants for one survey and 322 for the other.

### 2.2. Study Participants

The study surveyed a total of 657 participants, consisting of two groups: 328 parents of preschool and school-aged children, and 329 university and secondary school students. The only exclusion criterion was inappropriate age, with university and secondary school students under 18 years of age and preschool and school-aged children above 14 years of age being excluded. After applying the exclusion criteria, the first sub-sample consisted of 324 parents of preschool and school-aged children, while the second included 325 university and secondary school students, with a total sample size of 649 participants.

All participants were fully informed about the study’s objectives, benefits, and potential risks, and also that completing the questionnaire constituted their informed consent to participate in the study. The study was approved by the Ethics Committee of the University Department of Health Studies at the University of Split (Class: 029-03/24-18/01) and was conducted in full accordance with the General Data Protection Regulation.

### 2.3. Measurements

The survey for parents of preschool and school-aged children comprised 26 items, while the survey for university and secondary school students consisted of 23 items (a detailed description is provided in our previous publication [15]). Both surveys began with a section collecting general demographic data, including gender, age, height, weight, and educational level for both parents and their children, or for the students themselves.

Both surveys assessed participants’ habits of food label reading and their perception of the harmfulness of artificial sweeteners, energy drinks, and protein drinks/supplements. In addition, questions were included on snack and juice consumption during screen time (television, gaming, and computer use), and intake of various beverages, dairy-based protein drinks, and fruit yogurts. University and secondary school students were also specifically asked about their consumption of energy drinks and protein supplements (such as whey protein).

Participants were asked to list the specific brands of food products they most frequently consume, along with the quantity and frequency of consumption. The analysis of 121 food products was conducted to identify the presence of four artificial sweeteners in the most commonly reported ones: K-acesulfam, cyclamate, aspartame, and saccharin dihydrate. A significant portion of the sample consisted of various types of beverages, as these have been identified in multiple studies as major contributors to weight disturbances, with part of the sugar content replaced by artificial sweeteners [16]. Both carbonated and non-carbonated drinks, fruit-based beverages, and products used to prepare non-alcoholic drinks (such as syrups and instant products) were examined (in total 89). Among these, 60 (67.4%) contained one or more artificial sweeteners. The most commonly found sweetener was acesulfame K, present in 37 (61.7%) of the drinks, followed by cyclamate in 22 (36.7%), aspartame in 19 (31.7%), and saccharin in 18 (30.0%) of the beverages. Detailed results are provided in our paper, which is currently under publication [17].

### 2.4. Statistical Analysis

All categorical variables were presented as absolute numbers and percentages. All numerical variables were summarized using medians and interquartile ranges (IQRs), due to non-normal distribution, which was verified with the Kolmogorov–Smirnov test. Differences between groups were analyzed using the Chi-square test for categorical variables. One-sample Chi-square test was used to assess the distribution of responses across categories for each item (artificial sweeteners, energy drinks, and protein drinks), while a two-sample Chi-square test was used to compare distributions of responses between two distinct groups. Correlations between variables related to the consumption of various beverages or food items were evaluated using Spearman’s rank correlation test. A multivariate linear regression model was employed to assess the association between BMI and various participant characteristics. The main predictor variables included age, gender, sports activity, and variables related to food and beverage consumption. Beta coefficients with 95% confidence interval (CI) and *p*-values were reported for each variable included in the model.

The significance level was set at *p* < 0.05 (two-sided). Statistical analyses were performed using the Statistical Package for Social Science software, version 26 (SPSS Inc., Chicago, IL, USA).

## 3. Results

### 3.1. Demographic Data

Demographic characteristics of both parents and children for the first survey are detailed in a previous publication [15]. However, for consistency in presentation, the demographic data of the mentioned sample are also provided here (Table 1).

The majority of respondents were mothers (n = 288, 88.9%), and the average age of parents was 37.1 years. Educational attainment was high, as 51.9% of parents held a university degree, and 7.7% had completed a master’s or doctoral degree. The median BMI for parents was 22.94, while for children, it was 15.38 (Table 1).

The demographic characteristics of university and secondary school students are outlined in Table 2.

The demographic data of all participants are presented in Table 3.

The total sample for both surveys comprised 649 participants, with 40.2% female and 59.8% male participants. The median age was 18.0 years. The median BMI value was 18.9, with a range from 11.73 to 37.58. In terms of physical activity, 34.4% of participants reported engaging in physical activity three or more times per week, 21.0% exercised twice a week, 8.5% once a week, and 36.2% did not engage in any physical activity (Table 3).

### 3.2. Perceptions of Artificial Sweeteners in Food Products

A total of 59.3% of parents reported being fully aware that sugars in many fruit juices, soft drinks, and protein drinks are often replaced with artificial sweeteners. When asked about the perceived harm of these substances, 74.1% of parents believed artificial sweeteners to be harmful, 92.0% regarded energy drinks as harmful, and 45.7% considered protein drinks to be harmful (Table 4).

A total of 43.5% of parents reported sometimes reading product labels, 32.7% indicated that they usually do, and 16.0% stated that they always read the labels. Meanwhile, 7.7% of parents mentioned that they never read food product labels.

A total of 88.9% of university and secondary school student participants are fully aware that sugars in many fruit juices, soft drinks, and protein drinks have been replaced with artificial sweeteners. Regarding the perceived harm of these substances, 54.2% believe artificial sweeteners are harmful, 80.9% consider energy drinks harmful, and 18.8% view protein drinks as harmful (Table 5).

A total of 8% of university and secondary school student participants always read product labels, 21.3% read them mostly or frequently, 42.8% read them occasionally, and 28.0% reported that they never read food product labels. Additionally, 25.2% of participants stated that they choose products with reduced fat content, while 74.8% do not consider this factor when making their choices.

In the total sample, the majority of participants perceived artificial sweeteners (64.1%) and energy drinks (86.4%) as harmful, while protein drinks were mostly viewed as neutral (38.1%). Significant differences in responses were observed: *p* < 0.001 for artificial sweeteners and energy drinks, and *p* = 0.029 for protein drinks (Table 6), based on a one-sample Chi-square test.

**Figure 1:**
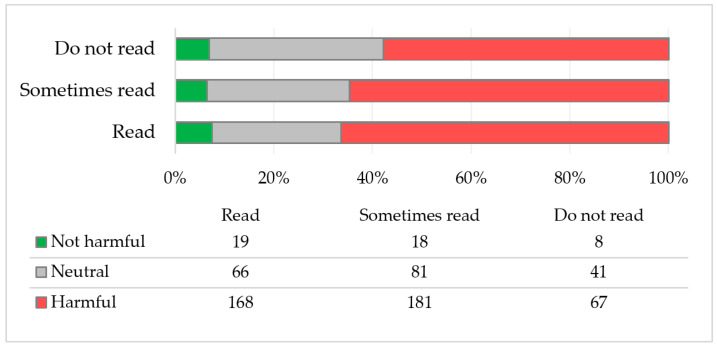
Perception of the harmfulness of artificial sweeteners in relation to the frequency of reading product labels (N = 649).

The majority of respondents hold a negative attitude towards artificial sweeteners, regardless of whether they read product labels or not. However, no statistically significant association was found between perceptions of the harmfulness of artificial sweeteners and the frequency of reading product labels (χ^2^ = 3.53; *p* = 0.474) (Figure 1).

**Figure 2:**
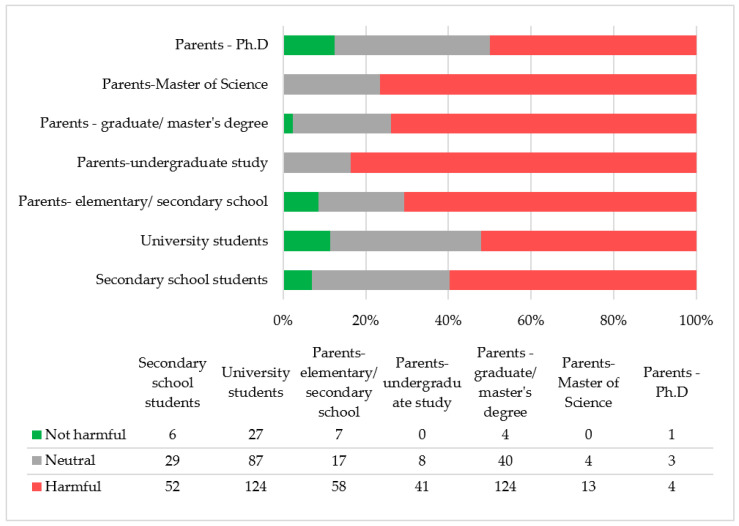
Perception of the harmfulness of artificial sweeteners among parents of preschool and school-aged children (categorized by level of formal education) as well as university and secondary school students (N = 649).

A statistically significant association (χ^2^ = 41.00, *p* < 0.001) was observed between formal education level and the perception of artificial sweeteners among parents of preschool and school-aged children, as well as university and secondary school students (Figure 2), indicating that perceptions of artificial sweeteners vary significantly based on education level. This difference may result from the distribution of education levels within the sample or specific variations in attitudes between parents and students.

However, among parents and students analyzed separately, no statistically significant association was found between education level and perceptions of the harmfulness of artificial sweeteners (χ^2^ = 13.35, *p* = 0.101, and χ^2^ = 2.19, *p* = 0.347, respectively).

### 3.3. Consumption of Various Food Products Containing Artificial Sweeteners

According to participants’ responses, only 0.9% always consume snacks and juices during screen time (watching TV, playing video games, or using a computer), while 13.4% indicated they do so. The majority (56.4%) engage in this behavior occasionally, while 29.3% stated that they never partake in it.

The frequency of consumption of various food products containing artificial sweeteners for the entire sample is presented in Table 7, with the breakdown for the two sub-samples available in the Supplementary Materials (Tables S1 and S2).

Additionally, the correlation coefficients between the consumption of various food products for the entire sample are provided in the Supplementary Materials (Table S3). A moderately strong and statistically significant positive relationship (highlighted by the two darkest shades of green) was observed between the consumption of different beverages and preparations. For water consumption, the correlation was negative, which is considered a positive outcome. However, the coefficient was not sufficiently strong and was generally not statistically significant (Supplementary Table S3).

According to parents’ reports, 55.6% of children sometimes eat snacks and drink juices during screen time, while 39.2% never engage in these behaviors. A smaller group of children (4.9%, n = 16) frequently consume snacks or juices during these activities, with only two children always consuming them.

Among university and secondary school students, the largest group of respondents (57.2%) reported sometimes eating snacks and drinking juices during screen time. An additional 21.8% indicated that they frequently engage in this behavior, 1.5% always, while 19.4% never partake in this habit.

### 3.4. Multivariate Analysis—Characteristics Associated with BMI

Multivariate linear regression identified several statistically significant associations with BMI. Male participants were found to have a higher BMI compared to females (β = 1.17; 95% CI 0.71–1.62; *p* < 0.001). Age was also positively associated with BMI (β = 0.42; 95% CI 0.39–0.45; *p* < 0.001). Participants who consumed carbonated soft drinks rarely or never exhibited a lower BMI (β = −1.48; 95% CI −2.88–−0.08; *p* = 0.039) compared to those who consumed them daily. Furthermore, occasional snack consumption was positively associated with BMI (β = 0.51; 95% CI 0.03–1.00; *p* = 0.039) when compared to those who did not consume snacks. The regression model demonstrated a good fit to the data (Durbin–Watson = 1.918; adjusted R^2^ = 0.649) (Table 8).

## 4. Discussion

### 4.1. Perceptions of the Harmfulness of Artificial Sweeteners

Overall, a comparison of the results from both groups revealed that parents of preschool and school-aged children held more negative attitudes towards artificial sweeteners than adults, including university and secondary school students. Specifically, 74.1% of parents believed that artificial sweeteners were harmful, compared to 54.2% of university and secondary school students. Negative perceptions of energy drinks were also more prevalent among parents (92.0%) than among university and secondary school students (80.9%). Similarly, 45.7% of parents expressed concerns about protein drinks, whereas only 18.8% of students shared this view. These findings align with the study by Farhat et al., which examined the knowledge and perceptions of artificial (non-nutritive) sweeteners among 1589 adults in the United Kingdom [18]. A significant proportion of participants in their study perceived artificial sweeteners as harmful, despite regression analysis indicating a correlation between perceived risk and actual consumption. Consistent with our results, their study also found that the perception of artificial sweeteners as harmful increased with age.

The results of a Spanish study, which included 100 participants each from the consumer, non-consumer, and healthcare professional groups, revealed a significant difference in beliefs regarding the consumption of non-nutritive sweeteners (NNSs). Healthcare professionals generally held negative perceptions of NNSs, believing their use should be limited to specific conditions and certain patients. In contrast, consumers had a positive view, while non-consumers remained neutral [19]. Similarly, our study found variations in overall attitudes toward artificial sweeteners with respect to formal education level. However, in this study, parental perceptions toward artificial sweeteners were not associated with their level of formal education. Among parents with a doctoral degree, 50.0% had negative perceptions, while 37.5% remained neutral. Across other educational levels, negative perceptions ranged from 52.1% to 83.7%. Notably, the highest positive perception (12.5%) was observed among participants with a doctoral degree, the highest level of education. Furthermore, university and secondary school students exhibited comparable attitudes, highlighting the complexity of public perceptions of artificial sweeteners across different demographic groups.

Supporting these findings, a study conducted in the United Kingdom examined the relationship between attitudes toward sugar intake, low-calorie sweeteners, and sweet-tasting foods among 581 participants. No clear correlation was found between formal education and attitudes or knowledge about sugar consumption. However, higher educational attainment was often associated with healthier eating habits [20]. These results are consistent with those reported in other studies [21,22,23,24] further highlighting the complexity of consumer perceptions regarding sweeteners, suggesting that attitudes are shaped by a combination of awareness, personal preferences, and broader cultural factors.

For instance, a survey of 1000 young adults in Canada revealed that 63.9% perceived high fructose corn syrup as unhealthier than aspartame or sucralose [25], highlighting variations in consumer perceptions of different sweeteners. Similarly, another study in the United Kingdom identified the primary motivation for consuming artificially sweetened products as their low energy content [18]. Notably, individuals who were overweight or obese perceived the consumption of such products as personally beneficial.

Moreover, studies revealed that educational interventions could shift consumer attitudes. After receiving information about artificial sweeteners, 33% of participants changed their view from positive to negative, while 44% admitted they had previously been unaware of the potential risks associated with these sweeteners [17]. These findings underscore the importance of targeted consumer education in shaping informed dietary choices, particularly in addressing misconceptions and raising awareness about the potential health implications of artificial sweeteners.

While our study did not identify a statistically significant relationship between attitudes toward the harmfulness of artificial sweeteners and the frequency of product label reading, the overall low engagement with label reading is noteworthy. Among parents of preschool and school-aged children, only 16.0% reported always reading labels, while 7.7% stated they occasionally do so. Among adult participants, only 8% reported always reading labels, while 28.0% indicated they never read labels at all. This low engagement in label reading is consistent with findings from a previous study assessing consumers’ opinions and use of food labels [26], which also found a statistical difference in the frequency of label reading based on gender, but not on education level or BMI. These findings suggest that limited attention to product labels may contribute to a lack of awareness regarding the presence and potential effects of artificial sweeteners in food products. Studies have shown that many consumers are unaware of the presence of artificial sweeteners in products [17].

Inconsistent food labeling practices further compound the issue. A scoping review revealed that the prevalence of declaring sweeteners on food labels ranged from less than 1% to 43.6%, emphasizing inconsistencies in labeling practices [27]. Manufacturers have leveraged the information about low-calorie content by highlighting the reduced caloric value of their products, while simultaneously omitting the presence of artificial sweeteners. Enhancing consumer education on the importance of reading labels could improve understanding and support more informed dietary choices.

Supporting this need for education, studies from other countries have shown varying levels of label reading and awareness. For instance, a study of Lebanese consumers including 768 participants revealed that nearly half (46.5%) regularly read labels, with 44.3% specifically checking for sugar content [28]. Similarly, a study in Kolkata involving 800 undergraduate medical students found inconsistent knowledge of artificial sweeteners. While participants expressed moderate concern, only 59.9% reported reading labels [29]. Additionally, market research from the Republic of Croatia also indicated that many average consumers with no health concerns typically do not read labels, leaving them unaware of what they are consuming [30,31]. The low rates of label reading observed in our study further underscore the need for consumer education. Survey results indicated that 13.2% of parents reported their children consumed dairy protein drinks weekly or daily, with this figure significantly higher among adults, at 39.7%. The analysis revealed that one of the dairy protein drinks frequently consumed by participants across all age groups contained the highest concentration of acesulfame K (352 mg L^−1^) [17]. Despite expressing negative views on the harmfulness of artificial sweeteners, the findings suggest that poor consumer awareness may contribute to increased consumption of products containing these sweeteners, potentially intensifying their negative health effects.

Given that perceptions and consumption trends regarding artificial sweeteners vary across countries, each new study offers valuable insights for developing effective communication and educational strategies to inform the public.

### 4.2. Consumption of Various Food Products and BMI

Research consistently demonstrates that parents play a fundamental role in shaping their children’s eating behaviors through both genetic influences and the home environment. During early childhood, as children transition from a milk-based diet to a more varied one, parental guidance plays a crucial role in shaping food preferences and dietary habits [32]. One key aspect of this influence is the practice of family meals, which has been associated with healthier eating habits in children. Regular family meals encourage the consumption of nutrient-rich foods like fruits, vegetables, and whole grains and are associated with a lower risk of obesity among children.

In our study, a total of 281 parents reported that they typically have two or more meals a day with their children, suggesting that children predominantly eat at home, where parents play a crucial role in shaping their children’s eating habits. Beyond meal frequency, the quality of parental consumption behaviors further impacts children’s health outcomes. Research on the intergenerational transmission of eating behaviors supports this claim. A large prospective cohort study in Denmark revealed that children whose mothers consumed sugar-sweetened and artificially sweetened beverages daily during pregnancy were 1.93 times more likely to be overweight or obese by the time they reached 7 years old [33]. This highlights the dual importance of parental consumption habits and shared family meals in shaping children’s long-term dietary patterns and health trajectories.

We found that the median BMI value was 15.38 among the preschool and school-aged children. A small difference in BMI values was observed between the two subgroups: for children aged 6.9 years and younger, the median was slightly lower (15.28), compared to those aged 7–14 years (15.93). Remarkably, 83% of the children had BMI values below the recommended range set by the WHO (18.5–24.9) [34], which could indicate undernourishment. Fifty-two children had a BMI within the recommended range, while only one child had a BMI above 24.9; specifically, their BMI was 28.3 (99.9th percentile). According to their responses, this child consumed 9 glasses of various juices and/or drinks and only 1 glass of water. Notably, the child’s parent also had a high BMI (BMI = 30), which is above the upper limit of the recommended range. However, it is important to mention that the Croatian population, unfortunately, tends to have higher average BMI values [14].

To more accurately assess the children’s nutritional status, the Pedi Tool software [35] was used to convert BMI values into percentiles. This analysis revealed that nearly 70% of participants had a normal body weight, while 7.6% were classified as overweight, 6.9% as obese, and 16% as undernourished. It is worth noting that the undernourished group may be impacted by the children’s rapid growth.

While artificial sweeteners have no caloric value, their frequent consumption can lead to increased food cravings, creating an energy imbalance between intake and expenditure. Children with low physical activity levels are especially susceptible to weight gain. However, our study found an unexpected disparity between BMI and physical activity levels, showing no significant association between BMI and any type of physical activity. This lack of association may be attributed to poor dietary habits, which can play a more prominent role in weight management. While BMI is a widely used indicator of nutritional status, it does not directly assess body composition, such as subcutaneous fat or muscle mass, which are crucial factors in understanding a child’s overall health [36,37].

According to parents’ reports, preschool and school-aged children most frequently consume homemade fruit juices (19.1%) daily, followed by fruit yogurts (10.8%), milk-based protein drinks (7.7%), and fruit syrups for dilution (4.6%), aside from water. Weekly consumption patterns showed similar trends, with homemade fruit juices remaining the most commonly consumed beverage (46.0%).

In contrast, university and secondary school students showed different consumption patterns. The median BMI value for this group was 22.15. Aside from water, the most frequently consumed beverages were milk-based protein drinks (12.6% daily) and protein supplements (9.8%), followed by store-bought fruit juices with high fruit content (9.8%) and homemade fruit juices (8.0%). Weekly consumption patterns indicated a significant intake of carbonated soft drinks (44.0%) and fruit juices (41.0%).

With the exception of water and homemade fruit juices, nearly all reported beverages contained artificial sweeteners [17]. Regulations aimed at reducing sugar levels in non-alcoholic beverages have resulted in the widespread use of artificial sweeteners as substitutes [38]. According to the 2019 European Health Survey, Croatia ranks among the EU countries with a high average BMI [14]. Numerous scientific studies have reported a correlation between BMI and the frequent consumption of high-sugar products [39,40]. However, public education on artificial sweeteners has been largely overlooked. While these sweeteners are often promoted for their lower caloric value, scientific research has raised concerns about their potential negative health effects. Even the World Health Organization (WHO) advises against their use for weight management [41].

Building on these concerns, multivariate regression analysis demonstrated a significant association between BMI and factors such as gender, age, and the consumption of carbonated soft drinks and snacks. Male participants had a BMI of 1.17 points higher compared to female participants (*p* < 0.001), and BMI increased by 0.42 points for each additional year of age (*p* < 0.001). Furthermore, participants who rarely or never consumed carbonated soft drinks had a BMI 1.48 points lower than those who consumed them daily (*p* = 0.039). Similarly, those who consumed snacks occasionally had a BMI 0.51 points lower than those who ate them daily (*p* = 0.039).

The results are consistent with a study conducted in Peru, involving 1414 children under 8 years of age, which revealed the significant link between daily consumption of sugary beverages and a significant increase in body weight. The multivariate analysis revealed that children who consumed sugary drinks and snacks daily experienced an average weight increase of 2.29 kg (95% CI 0.62, 3.96) and 2.04 kg (95% CI 0.48, 3.60), respectively, compared to those who never consumed such products [40]. This further supports the notion that frequent consumption of sugar-sweetened beverages and snacks contributes to higher BMI and underscores the need for public health initiatives focused on reducing excessive sugar intake.

### 4.3. Implications for Public Health Initiatives

While the present study was conducted in Croatia, the findings have broader implications for public health initiatives beyond national boundaries. The observed consumer perceptions and consumption patterns reflect global trends influenced by health consciousness and the growing demand for sugar-free products. For instance, the European Food Sweetener Market is expected to register a compound annual growth rate of 1.46% by 2030, driven by increasing consumer awareness and demand for low-calorie products [42]. Similarly, in Croatia, the sweeteners market is projected to grow by 4.32% between 2025 and 2030 [43], indicating that shifts in consumer behavior are part of a wider global movement.

To address these changing consumer preferences, legislative measures such as the Law on Special Tax on Coffee and Soft Drinks (NN 72/13, 121/19, 22/20) [44] have been implemented in Croatia to encourage the production of healthier products. This law introduces a sugar tax aimed at reducing added sugar content while encouraging manufacturers to increase the proportion of fruit juice in soft drinks. Specifically, it states that: “For fruit nectars and beverages under tariff heading KN 2009 that contain added sugars, the amount of special tax determined based on the sugar content is reduced by the percentage of fruit content present in the fruit nectar or beverage.” By promoting healthier product formulations, these regulatory initiatives align with broader public health objectives and support informed consumer choices.

Evidence from other countries further supports the effectiveness of such fiscal policy measures. For instance, similar legislative interventions in Poland have led to significant improvements in the quality of soft drinks, demonstrating the potential positive impact of fiscal policies on public health outcomes [45]. These examples illustrate how strategic policy actions can shape consumer behavior and drive industry changes, reinforcing the relevance of this study’s findings in a wider geographical context.

Taken together, this insight suggests that the drivers of consumer choices, such as health consciousness and marketing strategies, are not unique to Croatia but reflect broader global trends. By situating our results within this international framework, the study not only enhances the generalizability of the findings but also provides valuable insights for public health initiatives and nutrition education programs worldwide. This comparative perspective supports the development of targeted strategies to promote informed dietary choices. Consequently, future research should explore cross-cultural comparisons to better understand the influence of market dynamics on consumer behavior and to develop effective public health messaging strategies tailored to diverse populations.

### 4.4. Study Strengths and Limitations

The strength of this study lies in its comprehensive approach, which examines both participants’ perceptions of artificial sweeteners while also analyzing reported food products containing these additives, in both univariate and multivariate analysis. To the best of our knowledge, this is the first study of its kind conducted in Croatia. By utilizing a representative sample of participants, this research enhances understanding of the importance of consumer education. Additionally, it contributes to raising awareness of healthy dietary habits and promotes informed food choices among the younger population. Moreover, the study highlights the significant role of parental influence in shaping children’s dietary habits and the potential impact of artificial sweeteners on food cravings and energy balance. The observed disparities in beverage consumption patterns between younger and older participants suggest differing dietary risks, further emphasizing the need for targeted nutritional education.

Despite its strengths, this study has certain limitations. The use of voluntary and anonymous surveys may introduce potential sampling bias, as individuals with specific attitudes toward artificial sweeteners might have been more inclined to participate. Consequently, the findings may not fully represent the general population’s perceptions and behaviors. Additionally, the reliance on self-reported data is subject to recall bias and social desirability bias, which could affect the accuracy of reported consumption frequencies and perceptions. Although self-reported measures are widely used in nutrition and behavioral research, they may not always accurately reflect actual consumption patterns.

Furthermore, the study did not account for other potential confounding variables, such as dietary intake, genetic predisposition, socioeconomic status, and underlying health conditions, which could influence both artificial sweetener consumption and BMI. The sampling predominantly involved parents and students, which may limit the generalizability of the findings to the broader population. Future studies should aim to recruit a more diverse and representative sample to enhance external validity.

Another limitation is the lack of precise data on the quantity and type of artificial sweetener-containing foods consumed by children and young people. Additionally, male participants were underrepresented compared to females. Finally, the cross-sectional design provides a snapshot of consumer perceptions and consumption patterns at a single point in time, precluding causal inferences about the impact of artificial sweeteners on BMI and health outcomes. Longitudinal studies or experimental designs would strengthen the evidence by allowing for a more definitive assessment of causality.

## 5. Conclusions

The findings of this study indicate that most participants hold a negative attitude toward artificial sweeteners, yet their awareness and engagement with food label reading remain low. Notably, parents of preschool and school-aged children expressed greater concerns about the potential harm of artificial sweeteners compared to university and high school students. However, no statistically significant relationship was found between attitudes toward artificial sweeteners and the frequency of reading product labels, suggesting a disconnect between perception and actual dietary awareness.

In terms of dietary habits, the study found that the consumption of various artificially sweetened food products—except for carbonated soft drinks—did not serve as a statistically significant predictor of BMI among participants. However, occasional snack consumption was identified as a positive predictor of BMI, while water consumption showed a negative correlation with the intake of other beverages. Although this correlation was not strong (−0.012 to −0.105) and mostly not statistically significant, it indicated a general trend where individuals who consumed more water tended to drink fewer other beverages.

Overall, this research underscores the urgent need for public health initiatives that promote informed dietary choices. Addressing misconceptions, raising food label reading practices, and encouraging healthier consumption habits through educational campaigns will be essential in mitigating potential risks associated with artificial sweeteners, particularly among younger populations, as they are more likely to be influenced by marketing and peer perceptions. Tailored educational programs in schools and community settings could effectively raise awareness and encourage healthier consumption habits. Furthermore, public health messages should be culturally relevant and accessible to diverse demographic groups to maximize their impact. In addition, public health strategies should encourage balanced consumption habits and promote a holistic approach to nutrition that considers overall dietary patterns rather than focusing solely on individual ingredients. Collaborating with healthcare professionals, educators, and community organizations can enhance the credibility and reach of these initiatives.

## Figures and Tables

**Table 1:** Demographic characteristics of preschool and school-aged children (N = 324).

		Total (N = 324)	Age 1–6.9 (n = 251)	Age 7–14 (n = 73)
Gender; n (%)				
	Female	145 (44.8)	108 (43.0)	37 (50.7)
	Male	179 (55.2)	143 (57.0)	36 (49.3)
Age; median (IQR)		5.00 (2.00)	5.00 (3.0)	9.00 (7.0)
BMI; median (IQR)		15.38 (2.20)	15.28 (2.03)	15.93 (2.89)
BMI parent; median (IQR)		22.94 (4.17)	22.77 (3.90)	23.08 (6.62)
Physical activity; n (%)				
	No	114 (35.2)	100 (39.8)	14 (19.2)
	Yes, once a week	13 (4.0)	11 (4.4)	2 (2.7)
	Yes, twice a week	84 (26.9)	67 (26.7)	17 (23.3)
Yes, 3 or more times a week		113 (34.9)	73 (29.1)	40 (54.8)
Family shared meals during the day; n (%)				
	0	2 (0.6)	0 (0.0)	2 (0.6)
	1	41 (12.7)	31 (12.4)	10 (13.7)
	2	136 (42.0)	108 (43.0)	28 (38.4)
	3	79 (24.4)	60 (23.9)	19 (26.0)
	≥4	66 (20.4)	52 (20.7)	14 (19.2)

BMI—body mass index; IQR—interquartile range.

**Table 2:** Demographic characteristics of university and secondary school students (N = 325).

		Total (N = 325)
Gender; n (%)		
	Female	243 (74.8)
	Male	82 (25.2)
Age; median (IQR)		21.00 (6.00)
BMI; median (IQR)		22.15 (4.10)
Meals during the day; n (%)		
1		4 (1.2)
2		63 (19.4)
3		163 (50.2)
More than 3		95 (29.2)
Physical activity; n (%)		
	No	121 (37.2)
	Yes, once a week	42 (12.9)
	Yes, twice a week	52 (16.0)
Yes, 3 or more times a week		110 (33.8)
Education; n (%)		
	Faculty of Biomedical Sciences	154 (47.4)
	Faculty of Natural Sciences	27 (8.3)
	Faculty of Social Sciences	16 (4.9)
	Faculty of Engineering	9 (2.8)
	Faculty from other fields	32 (9.8)
	Grammar secondary school	61 (18.8)
	Vocational secondary school	23 (7.1)
	Health vocational school	2 (0.6)
	other type of secondary school	1 (0.3)

BMI—body mass index; IQR—interquartile range.

**Table 3:** Demographic characteristics of the entire sample (N = 649).

		Total (N = 649)
Gender; n (%)		
	Female	261 (40.2)
	Male	388 (59.8)
Age; median (IQR)		18.00 (16.00)
BMI; median (IQR)		18.93 (6.98)
Physical activity; n (%)		
	No	235 (36.2)
	Yes, once a week	55 (8.5)
	Yes, twice a week	136 (21.0)
Yes, 3 or more times a week		223 (34.4)

BMI—body mass index; IQR—interquartile range.

**Table 4:** Parents’ perceptions of the harmfulness of consumption (N = 324).

	Artificial Sweeteners	Energy Drinks	Protein Drinks
Not harmful	12 (3.7%)	7 (2.2%)	54 (16.7%)
Neutral	72 (22.2%)	19 (5.9%)	122 (37.7%)
Harmful	240 (74.1%)	298 (92.0%)	148 (45.7%)
*p*-value *	<0.001	<0.001	<0.001

* Chi-square test.

**Table 5:** University and secondary school students’ perceptions of the harmfulness of consumption (N = 325).

	Artificial Sweeteners	Energy Drinks	Protein Drinks
Not harmful	33 (10.2%)	14 (4.3%)	139 (42.8%)
Neutral	116 (35.7%)	48 (14.8%)	125 (38.6%)
Harmful	176 (54.2%)	263 (80.9%)	61 (18.8%)
*p*-value *	<0.001	<0.001	<0.001

* Chi-square test.

**Table 6:** Perceptions of the harmfulness of consumption for all participants (N = 649).

	Artificial Sweeteners	Energy Drinks	Protein Drinks
Not harmful	45 (6.93%)	21 (3.24%)	193 (29.74%)
Neutral	188 (28.97%)	67 (10.32%)	247 (38.06%)
Harmful	416 (64.10%)	561 (86.44%)	209 (32.20%)
*p*-value *	<0.001	<0.001	0.029

* Chi-square test.

**Table 7:** Consumption of various types of food products (N = 649).

	Rarely or Never	Once a Week	2–3 Times a Week	Every Day	*p*-Value *
Store-bought fruit juices with a high fruit content; n (%)	386 (59.5)	105 (16.2)	118 (18.2)	40 (6.2)	<0.001
Water; n (%)	2 (0.3)	1 (0.2)	5 (0.8)	641 (98.9)	<0.001
Carbonated soft drinks; n (%)	446 (68.7)	113 (17.4)	69 (10.6)	21 (3.2)	<0.001
Powdered (instant) drinks; n (%)	486 (74.9)	60 (9.2)	73 (11.2)	30 (4.6)	<0.001
Fruit syrups for dilution; n (%)	546 (84.1)	31 (4.8)	46 (7.1)	26 (4.0)	<0.001
Isotonic drinks; n (%)	587 (90.4)	31 (4.8)	22 (3.4)	9 (1.4)	<0.001
Iced tea; n (%)	523 (80.6)	63 (9.7)	50 (7.7)	13 (2.0)	<0.001
Flavored water; n (%)	548 (84.4)	41 (6.3)	48 (7.4)	12 (1.8)	<0.001
Homemade fruit juices; n (%)	293 (45.1)	131 (20.2)	137 (21.1)	88 (13.6)	<0.001
Dairy-based protein drinks; n (%)	477 (73.5)	44 (6.8)	62 (9.6)	66 (10.2)	<0.001
Fruit yogurt; n (%)	357 (55.0)	112 (17.3)	131 (20.2)	49 (7.6)	<0.001

* Chi-square test.

**Table 8:** Characteristics associated with BMI, determined by linear regression (all independent variables included in the model simultaneously, N = 649).

	Beta (95% Confidence Interval); *p*-Value
Gender (Referent (Ref.) female)	
Male	1.17 (0.71, 1.62); <0.001
Age (years)	0.42 (0.39, 0.45); <0.001
Physical activity (Ref. no)	
Once a week	0.5 (−0.31, 1.31); 0.223
Twice a week	−0.1 (−0.66, 0.46); 0.726
Three times a week	−0.32 (−0.83, 0.19); 0.212
Consumption of powdered (instant) drinks (Ref. every day)	
Rarely or never	−0.22 (−1.33, 0.89); 0.695
Once a week	−0.18 (−1.47, 1.10); 0.779
Two or three times a week	0.24 (−1.00, 1.48); 0.703
Consumption of fruit syrups for dilution (Ref. every day)	
Rarely or never	−0.61 (−1.77, 0.54); 0.298
Once a week	−0.58 (−2.06, 0.90); 0.441
Two or three times a week	−1.05 (−2.4, 0.29); 0.125
Consumption of isotonic drinks (Ref. every day)	
Rarely or never	1.35 (−0.78, 3.48); 0.213
Once a week	0.77 (−1.54, 3.07); 0.514
Two or three times a week	0.65 (−1.70, 2.99); 0.589
Consumption of iced tea (Ref. every day)	
Rarely or never	−0.25 (−2.09, 1.58); 0.787
Once a week	−0.77 (−2.71, 1.17); 0.436
Two or three times a week	−0.66 (−2.59, 1.28); 0.506
Consumption of homemade fruit juices (Ref. every day)	
Rarely or never	0.12 (−0.55, 0.80); 0.722
Once a week	−0.17 (−0.93, 0.58); 0.657
Two or three times a week	−0.05 (−0.79, 0.68); 0.892
Consumption of carbonated soft drinks (Ref. every day)	
Rarely or never	−1.48 (−2.88, −0.08); 0.039
Once a week	−0.67 (−2.11, 0.77); 0.358
Two or three times a week	−0.52 (−1.97, 0.94); 0.489
Consumption of fruit yogurts (Ref. every day)	
Rarely or never	0.22 (−0.65, 1.09); 0.626
Once a week	0.15 (−0.81, 1.11); 0.756
Two or three times a week	0.30 (−0.63, 1.24); 0.522
Consumption of snacks (Ref. never)	
Always	−0.37 (−2.63, 1.89); 0.745
Frequently	−0.17 (−0.91, 0.58); 0.662
Sometimes	0.51 (0.03, 1.00); 0.039

## Data Availability

The data presented in this study are available upon request from the corresponding author.

## Supplementary Materials

The following supporting information can be downloaded at https://www.mdpi.com/article/10.3390/nu17050814/s1, Table S1: Consumption of food products among preschool and primary school children, reported by parents (N = 324); Table S2: Consumption of food products among university and secondary school students (N = 345); Table S3: Correlation between the various types of food products (N = 649).

## Abbreviations

The following abbreviations are used in this manuscript:

BMI	Body mass index
CI	Confidence interval
IQR	Interquartile range
NNSs	Non-nutritive sweeteners
WHO	Word Health Organization
