# Supplementary material for: Consumer Perceptions of Artificial Sweeteners in Food Products, Consumption Frequency, and Body Mass Index: A Multivariate Analysis
Source: Nutrients. 2025 Feb 27;17(5):814. doi: 10.3390/nu17050814 (PMC11902076; doi:10.3390/nu17050814)
Supplement: Supplementary file 1 [file nutrients-17-00814-s001.zip › nutrients-3498393-supplementary.pdf]

Table S1. Consumption of food products among preschool and primary school children, reported by parents (N=324).

	**rarely or never**	**once a week**	**2-3 times a week**	**every day**	***p*-value***
Store-bought fruit juices with a high fruit content; n (%)	226 (69.8)	46 (14.2)	44 (13.6)	8 (2.5)	<0.001
Water; n (%)	1 (0.03)	1 (0.03)	1 (0.03)	321 (99.1)	<0.001
Carbonated soft drinks; n (%)	282 (87.0)	25 (7.7)	14 (4.3)	3 (0.9)	<0.001
Powdered (instant) drinks; n (%)	259 (79.9)	22 (6.8)	34 (10.5)	9 (2.8)	<0.001
Fruit syrups for dilution; n (%)	262 (80.9)	17 (5.2)	30 (9.3)	15 (4.6)	<0.001
Isotonic drinks; n (%)	305 (94.1)	5 (1.5)	9 (2.8)	5 (1.5)	<0.001
Iced tea; n (%)	284 (87.7)	15 (4.6)	22 (6.8)	3 (0.9)	<0.001
Flavored water; n (%)	296 (91.4)	7 (2.2)	18 (5.6)	3 (0.9)	<0.001
Fruit juices without sugar; n(%)	258 (79.6)	26 (8.0)	35 (10.8)	5 (1.5)	<0.001
Homemade fruit juices; n (%)	113 (34.9)	61 (18.8)	88 (27.2)	62 (19.1)	<0.001
Dairy-based protein drinks; n (%)	281 (86.7)	2 (0.6)	16 (4.9)	25 (7.7)	<0.001
Fruit yogurt; n (%)	176 (54.3)	42 (13.0)	71 (21.9)	35 (10.8)	<0.001

*Chi-sqare test

Table S2. Consumption of food products among university and secondary school students (N=345).

	**rarely or never**	**once a week**	**2-3 times a week**	**every day**	***p*-value***
Store-bought fruit juices with a high fruit content; n (%)	160 (49.2)	59 (18.2)	74 (22.8)	32 (9.8)	<0.001
Water; n (%)	1 (0.3)	0 (0.0)	4 (1.2)	320 (98.5)	<0.001
Carbonated soft drinks; n (%)	164 (50.5)	88 (27.1)	55 (16.9)	18 (5.5)	<0.001
Powdered (instant) drinks; n (%)	227 (69.8)	38 (11.7)	39 (12.0)	21 (6.5)	<0.001
Fruit syrups for dilution; n (%)	284 (87.4)	14 (4.3)	16 (4.9)	11 (3.4)	<0.001
Isotonic drinks; n (%)	282 (86.8)	26 (8.0)	13 (4.0)	4 (1.2)	<0.001
Iced tea; n (%)	239 (73.5)	48 (14.8)	28 (8.6)	10 (3.1)	<0.001
Flavored water; n (%)	252 (77.5)	34 (10.5)	30 (9.2)	9 (2.8)	<0.001
Homemade fruit juices; n (%)	180 (55.4)	70 (21.5)	49 (15.1)	26 (8.0)	<0.001
Dairy-based protein drinks; n (%)	196 (60.3)	42 (12.9)	46 (14.2)	41 (12.6)	<0.001
Fruit yogurt; n (%)	181 (55.7)	70 (21.5)	60 (18.5)	14 (4.3)	<0.001
Energy drinks; n (%)	291 (89.5)	15 (4.6)	17 (5.2)	2 (0.6)	<0.001
Protein supplements (whey protein); n (%)	243 (74.8)	15 (4.6)	35 (10.8)	32 (9.8)	<0.001

*Chi-sqare test

Table S3. Correlation between the various types of food products (N=649).

	Store-bought fruit juices with a high fruit content	Water	Carbonated soft drinks	Powdered (instant) drinks	Fruit syrups for dilution	Isotonic drinks	Iced tea	Flavored water	Homemade fruit juices	Dairy-based protein drinks	Fruit yogurt
Store-bought fruit juices with a high fruit content	1.000	-0.049	0.411**	0.289**	0.226**	0.300**	0.368**	0.316**	0.042	0.140**	0.225**
Water		1.000	-0.023	-0.012	-0.028	-0.105**	-0.053	-0.067	0.043	-0.067	-0.048
Carbonated soft drinks			1.000	0.257**	0.214**	0.377**	0.327**	0.362**	-0.021	0.189**	0.140**
Powdered (instant) drinks				1.000	0.190**	0.320**	0.271**	0.143**	0.056	0.064	0.093*
Fruit syrups for dilution					1.000	0.271**	0.253**	0.183**	0.185**	-0.039	0.183**
Isotonic drinks						1.000	0.397**	0.426**	0.074	0.202**	0.154**
Iced tea							1.000	0.341**	0.126**	0.142**	0.172**
Flavored water								1.000	0.087*	0.169**	0.129**
Homemade fruit juices									1.000	-0.027	0.106**
Dairy-based protein drinks										1.000	0.131**
Fruit yogurt											1.000

**Correlation coefficient significant at the 0.01 level ($p < 0.010$).

*Correlation coefficient significant at the 0.05 level ($p < 0.050$).
